# Reasons and Reproduction: Gene Editing and Genetic Selection

**DOI:** 10.1080/15265161.2023.2250288

**Published:** 2023-09-11

**Authors:** Jeff McMahan, Julian Savulescu

**Affiliations:** a University of Oxford; b Yong Loo Lin School of Medicine, National University of Singapore; c Murdoch Children's Research Institute; d Melbourne Law School, University of Melbourne

**Keywords:** Genetic research, genetically modified organisms, reproductive technologies, philosophy, enhancement

## Abstract

Many writers in bioethics, science, and medicine contend that embryo selection is a morally better way of avoiding genetic disorders then gene editing, as the latter has risks that the former does not. We argue that one reason to use gene editing is that in many cases it would be *better* for the person who would develop from the edited embryo, so that not to have done it would have been *worse for* that person. By contrast, embryo selection is never better for the person who develops from the selected embryo. This reason to use gene editing has, however, been challenged on two grounds: first, that it makes no difference, morally, whether a bad effect is worse for someone, or a good effect better for someone; and, second, that beneficent gene editing would not be unequivocally better for the person who would develop from the edited embryo. We argue that both of these objections can be satisfactorily answered and thus that there is indeed a significant moral reason, at least in some cases, to use gene editing rather than embryo selection.

## THE NATURE OF REASONS AND THE METAPHYSICS OF REPRODUCTION

The dominant view in bioethics is that embryo selection is in general morally preferable to gene editing because the latter involves risks that are absent in the former. What is less widely appreciated is that the beneficent editing of an embryo’s genes can be and often is *better for* the person who develops from that embryo, while embryo selection is never foreseeably better for the person who develops from the selected embryo. This is because, whereas gene editing improves the condition of one and the same individual relative to certain alternatives, embryo selection only causes a better-off individual to exist rather than a different, less well-off individual. Although some deny that this difference is morally significant, we attempt to show, by exploring the implications of three rival views in population ethics, that it is indeed significant.

During the Zika epidemic in 2015-16, Public Health England (PHE) advised people returning from a Zika-infested area, such as Brazil, who were considering having a child to wait three months before doing so (Savulescu and Kahane 2016). By waiting, the potential parents could avoid any risk that the embryo or fetus would be affected by Zika, which could cause microcephaly and cognitive disability, which PHE assumed are bad and to be avoided.

This advice, when followed, would have resulted in a different embryo and thus a different child coming into existence. For if a couple wait three months to conceive a child, the sperm and egg that will join will be different from those that would have joined three months earlier. There is thus a roughly 50 percent chance that a child conceived three months later would be of a different sex.

We can refer to an embryo that a couple might have produced immediately on their return from Brazil as “Dom” and an embryo they might have produced three months later as “Hannah.” PHE’s advice was, in effect, that the couple should produce Hannah rather than Dom, because Hannah would probably have had no impairments, whereas Dom might have had serious cognitive impairments.

If the couple had waited three months, Dom would never have existed, which would not have been bad or worse for him. Hannah would have existed instead, which would have been good for her, assuming that her life would have been worth living. But it would not have been *better* for her. This is because “better” and “worse” are comparative terms. Thus the claim that Hannah’s coming into existence would have been *better* for her implies that her never coming into existence would have been *worse* for her. But if Hannah had never existed, that could not have been worse for her. There cannot be anyone for whom never existing is worse than existing would have been.

If instead the couple had conceived a child immediately on their return, Hannah would never have existed but Dom would have. Suppose that Dom would have suffered serious cognitive impairments because of the Zika virus. Even so, assuming his life would have been worth living, his parents’ action would not have been worse or even on balance bad for him, as the alternative was that he would never have existed. Indeed, it would have been on balance *good* (though not *better*) for him. So, whichever child the couple had had, that would have been on balance good for that child and neither bad nor worse for the other possible child. Still, it seems that PHE was right that the couple had a strong moral reason to wait three months and cause Hannah to exist rather than Dom.

As these remarks indicate, we cannot explain the reason to cause the better-off person to exist rather than a different, less well-off person in what Derek Parfit called “narrow person-affecting” terms—that is, in terms of effects on people for *better* or *worse*. At least in his earlier work, Parfit argued that the reason to cause a better-off person to exist rather than a different, less well-off person is “impersonal,” by which he meant that it is concerned with what is good or bad in itself, which might not be better or worse, or even good or bad, for any individual (Parfit 1987). He also recognized that, assuming that it can be good or bad for a person to be caused to exist, there could be “wide person-affecting” reasons, which are reasons to do what would be good, though not better, for people and not to do what would be bad, though not worse, for people. As these reasons might generally coincide with impersonal reasons that are concerned with well-being and ill-being, we will not discuss them here, though we actually think that reasons to cause or not to cause people to exist are more plausibly understood as wide person-affecting than as impersonal, as indeed Parfit himself came to believe later in his life (Parfit 2017).

We can now distinguish three views about the nature of the moral reasons that are concerned with well-being and ill-being and, in particular, the reasons that govern choices about causing or not causing people to exist.

### The Comparative View

Reasons to promote well-being and to prevent ill-being are narrow person-affecting (henceforth simply “person-affecting”). On this view, benefiting and harming are *comparative*: to benefit a person is to do what is better for that person and to harm a person is to do what is worse for that person. One cannot, therefore, benefit or harm a person by causing that person to exist.

### The Impersonal View

Reasons to promote well-being and to prevent ill-being are impersonal in the sense that they are reasons to produce outcomes in which there would be more well-being, on balance, or in which well-being would be better distributed, or both, than in other possible outcomes. This is true irrespective of whether the impersonally best outcome would be better for anyone.

### The Two-Tier View

There are both person-affecting and impersonal reasons. There is thus (1) a reason to do what would be better for a person, (2) a reason not to do what would be worse for a person, (3) a reason to cause a better-off person to exist rather than cause or allow a different, less well-off person to exist, (4) a reason not to cause a miserable person—that is, a person whose life is overall bad for her or below the neutral level for well-being—to exist, and (5) a reason to cause a well-off person to exist rather than not cause anyone to exist. But these reasons are not equal in strength, even if the amounts of well-being or ill-being are the same in each case. In general, person-affecting reasons are stronger than corresponding impersonal reasons. Thus, a reason of type 1 is stronger than a reason of type 3, even if the difference in well-being is the same in each case. And the reason to do what is better for a person—for example, by saving that person’s life—is stronger than the reason to cause a well-off person to exist, even if the net well-being one enables the beneficiary to have would be the same in both cases. (Most of us believe that there is a moral asymmetry between reasons concerned with well-being and those concerned with ill-being, and hence believe that the reason not to cause a miserable person to exist is not much weaker, if it is weaker at all, than the reason not to cause an existing person to suffer equivalent misery). While it seems that there must be both a person-affecting reason and an impersonal reason to benefit an existing person, these reasons are not additive: the person-affecting reason is stronger and simply displaces the impersonal reason.

To assess the relative plausibility of these three views, we should explore their implications for various reproductive and other choices.

## DELIBERATELY CREATING DEAF CHILDREN

Some people—both deaf people and others—view deafness as a mere difference, not a disadvantage in itself (Barnes 2014). They argue that the deaf community constitutes a cultural minority and that sign language is a unique and effective form of communication. Many deaf people take pride in their culture and sometimes wish to have a deaf child rather than a child who can hear (Savulescu 2002).

There are three ways a couple could deliberately have a deaf child:

*Selection*: a couple could deliberately produce a number of embryos via IVF and select one that would be deaf (or procure gametes from other deaf people).
*Refusal of treatment*: parents could refuse cochlear implants for their deaf child.
*Deafening*: an embryo could have its genes edited to make it deaf, an infant’s auditory nerves could be cut, or an infant could be administered drugs that would cause it to become deaf.

As far as we know, the last type of procedure has not been done, but it has much the same effect as the refusal of treatment. Both these last two means of producing deafness have outcomes that are similar in person-affecting terms. The main difference is that deafening intentionally *causes* an individual to be deaf, whereas refusal of treatment intentionally *allows* an individual to be deaf.

We can compare two of these ways of *causing* deafness: selecting a deaf embryo and editing the genes of a hearing embryo to produce deafness by introducing the same mutation that causes deafness in the selected embryo. To understand the possible moral differences between these two ways of causing deafness, we need to be aware of two relevant metaphysical issues. One is whether an early embryo would be one and the same individual as the person who might develop from it—that is, whether we were once early embryos. There are, of course, different views about when we begin to exist. If we begin to exist before gene editing might be done, then it is clear that the editing can be better or worse for the person who may develop from the embryo—for that same individual already exists when the editing is done. And it is highly unlikely that an alteration to an embryo’s genes would cause that embryo to cease to exist and be replaced by a different embryo. But if the same embryo continues to exist through the process of gene editing, and if that embryo is one and the same individual as the person who might develop from it, then the same person will exist whether or not the editing is done. Also, on the assumption that we already exist when gene editing might be done, the discarding of an early embryo is the killing of one of us when that individual was very young.

If, however, we do not begin to exist until some time after gene editing might be done—that is, if we were never early embryos—then a highly important question arises. We know that a specific person will develop from a particular early embryo if that embryo is implanted unaltered and develops normally. It seems, however, that if the genes of an early embryo are radically altered, the person who will develop from it will be different from the person who would have developed in the absence of the alteration. Radical alterations would, we can say, be “identity-determining.” If, for example, we are essentially human organisms, it seems that an alteration that would change the embryo’s biological sex would cause a different person to come into existence. When gene editing is identity-determining, it is relevantly like embryo selection in that it causes one person to come into existence rather than another.

But on any plausible account of our identity, it seems that a trivial genetic alteration, such as one that results in only a slight change in the shade of the eye color, would be insufficient to cause the existence of a different person. It would instead be “identity-preserving.”

A genetic alteration that would produce deafness only is intermediate between these examples of identity-determining and identity-preserving alterations. To us it seems reasonable to suppose that, on any plausible account of our identity, an alteration causing deafness only would be identity-preserving. But we are unable to provide a defense of a general theory about this metaphysical issue that supports this view. We therefore ask the reader to grant for the sake of argument the assumption that gene editing to produce deafness only would be identity-preserving.

We will not here enter into the debate about whether deafness is a disadvantageous condition. Although one of us has argued that deafness is not intrinsically bad and on the whole not nearly as bad as most hearing people suppose (Savulescu and Kahane 2009), we will assume, for the sake of argument, that it is normally disadvantageous. This is useful for the purpose of illustration because most people believe deafness is disadvantageous, yet some deaf people want their children to be deaf.

Because the selection of a deaf embryo rather than a hearing embryo will not be *worse for* the person who develops from the selected embryo, and would indeed be *good* (though not better) for that person, the moral reason to select a hearing rather than a deaf embryo cannot be person-affecting. It must instead be impersonal. It is grounded, not in the well-being of the child who will be deaf, but only in the comparison between that child and a different possible child who might have existed and been better off. This supports the view that the selection of a deaf embryo should be legally permissible even in those cases in which it is morally wrong, all things considered (Savulescu 2002). Selection of a deaf embryo can reasonably be understood as a victimless wrong.

We can now compare the implications of the three moral views distinguished earlier for the morality of causing deafness through gene editing and embryo selection. On the assumption that deafness is normally a disadvantageous condition, all things considered, the Comparative View implies that causing deafness by gene editing would be wrong, other things being equal, as it would likely be worse for the person who would develop from the edited embryo. Yet there would be nothing wrong, other things being equal, in selecting a deaf embryo rather than a hearing embryo, as that would not be worse for the person who would develop from the selected embryo. This seems, however, to attribute too much significance to the relevant difference between gene editing and embryo selection. Indeed, because causing a person to exist rather than not doing so can never be better or worse for that person, the Comparative View implies that there would also be nothing wrong, other things being equal, in causing a less well-off person to exist rather a different, better-off person, or even in causing a person to exist whose life would be utterly miserable and not worth living (Parfit 1987). That it has these implications is, we will assume, a sufficient reason to reject the Comparative View.

The Impersonal View, by contrast, can recognize that—and give a reason why—it is wrong, other things being equal, to select a deaf embryo rather than a hearing embryo. It can also recognize that it is wrong to use gene editing to cause the person who develops from the edited embryo to be deaf. It implies, however, that both these means of causing deafness are equally objectionable, which seems implausible given that the selection of the deaf embryo would be *good for* (though, again, not better for) the person who would develop from the selected embryo, whereas causing deafness by gene editing would be *worse for* the person who develops from the edited embryo, as that person would otherwise have been able to hear.

The Impersonal View also implies that there is, in general, a reason to cause a better-off person to exist rather than a different, less well-off person, and also a strong reason not to cause a miserable person to exist. Yet the explanation it offers of this latter claim—namely, that to cause a miserable person to exist makes the outcome worse—seems at best only a partial explanation. The fundamental explanation seems to be that to cause such a person to exist would be terribly bad for, and thus terribly harmful to, that particular person.

Finally, just as the Impersonal View implies that there is a reason not to cause a miserable person to exist that is proportional in strength to the extent to which the life would be miserable, so it also seems to imply that there is a reason to cause a well-off person to exist that is proportional in strength to the extent to which the life would be good. (It is more difficult, theoretically, to defend the view that the infliction of a harm is worse, other things being equal, than the failure to confer an equivalent benefit, if one accepts the Impersonal View.) On the Impersonal View, then, the reason to cause a person to exist rather than not cause anyone to exist will in general be stronger than the reason to save a person’s life, as creating an entire good life will normally make the outcome better by more than preserving the remainder of a good life would. That it has these implications seems to be a sufficient reason to reject the Impersonal View.

This leaves the Two-Tier View. Because this view accepts that there are both person-affecting and impersonal reasons, it implies the more plausible conclusions of the other two views while avoiding their more extreme implications. According to the Two-Tier View, while there is, in general, a moral reason to select a hearing embryo rather than a different, deaf embryo, that reason is impersonal and is thus weaker than the person-affecting reason not to use gene editing to cause a person to be deaf (or to deafen a hearing infant), which would be worse for that person. This seems intuitively plausible. And it provides support for the view that, although it should be legally permissible to select a deaf embryo for implantation, it should be illegal to deafen an infant or to modify an embryo or fetus to cause deafness. There is also, as we noted, an intermediate option, which is for parents to refuse a cochlear implant for their deaf child. This is legally permissible in some states in the US. It is morally intermediate, on a deontological understanding of the Two-Tier View, because it involves intentionally *allowing* children to remain in a condition that is likely to be worse for them rather than *causing* them to be in that condition.

Like the Impersonal View, the Two-Tier View accepts that there are reasons not to cause miserable people to exist as well as reasons to cause better-off people to exist rather than different, less well-off people. Yet because the Two-Tier View accepts that there are both person-affecting and impersonal reasons, it too implies that there is a moral reason to cause a well-off person to exist rather than not cause anyone to exist. This may seem counterintuitive; but the view can appeal to the moral asymmetry, noted earlier, between reasons concerned with well-being and those concerned with ill-being as a basis for claiming that the reason not to cause a miserable person to exist is significantly stronger than the reason to cause a well-off person to exist. And because it holds that person-affecting reasons are stronger than corresponding impersonal reasons, it can avoid the implication of the Impersonal View that the reason to cause a person to exist is generally as strong as, or even stronger than, the reason to save a person’s life.

We can next compare the use of embryo selection and gene editing as means, not of *causing* genetic disorders, but of *avoiding* them. Consider, for example, what the three views we have canvased imply about the avoidance of cystic fibrosis (CF) through embryo selection and through gene editing. We will assume that it is highly likely that a life with CF would be less good than the same life would be without it, if other things are equal, and that editing out the gene for CF would be identity-preserving.

According to the Comparative View, there is strong reason to edit out the gene that causes CF, thereby enabling a person to have a life without CF rather than a life with it. But there is no reason, apart from reasons stemming from effects on others, to select a healthy embryo rather than one with CF. According to the Impersonal View, there is a strong reason to edit the genes of an embryo to prevent the person who would develop from it from having CF and an equally strong reason to select a healthy embryo rather than one with CF. Parfit draws the same conclusion about a relevantly similar comparison in an example he calls “the Medical Programmes” (Parfit 1987). He deploys this example to provide intuitive support for what he calls the “No-Difference View,” which is, in effect, the view that it makes no moral difference whether a bad effect is *worse* for someone or whether a good effect is *better* for someone.

But, while the selection of an embryo with the gene for CF rather than an otherwise similar embryo without it need not be worse for anyone, the failure to edit out the gene for CF would be worse for the person who would develop from the unedited embryo. According to the Two-Tier View, this difference matters morally. Thus, while there is a strong impersonal reason to select an embryo without the gene for CF, there is an even stronger person-affecting reason to edit this gene out in an embryo that has it, assuming that that embryo will later give rise to the existence of the same person either way. This is intuitively plausible, and supports the Two-Tier View.

## THE POPULAR POSITION, IMPERSONAL REASONS, AND COMMON PRACTICE

Many people in the popular media and science media claim, however, that embryo selection is morally preferable to gene editing (National Academy of Sciences et al 2017; Nuffield Council on Bioethics 2016). Indeed, some argue that we should never engage in gene editing. The main objection of the opponents of gene editing is that it involves risks—in particular, a risk of an unforeseen, harmful mutation—for the person whose genes have been edited. Embryo selection, by contrast, is simply a matter of causing one person to exist rather than another. There is no risk of causing harm to the person who will develop from the embryo that is selected, and—given our assumption that early embryos are not identical to later persons—no harm to those that are not selected. It is this contrast that prompts Marcy Darnovsky to claim that “embryo selection … is far less ethically fraught than manipulating the genes of future children” (Darnovsky 2019). We can refer to the view expressed here by Darnovsky as the “popular position.”

The Two-Tier View suggests that the popular position is mistaken. Because gene editing, when successful, enables people to avoid serious harms, it can be morally justifiable even when it involves risks (such as “off-target” mutations) for the individuals it is intended to benefit. It can be justified when the harms it is intended to prevent outweigh the possible harms it might cause, taking probabilities into account. In this respect it is like other medical treatments that promise benefits but also have a risk of causing harmful side effects.

How great could the risks permissibly be? There are various instruments for quantifying the value of risk and quality of life. For example, the quality of life measurements used to derive Quality-Adjusted Life Years can be derived from standard gamble (of death) or time trade-off. The quality of life for a person with moderately severe CF is estimated to be in the range of 0.8, where 1 is normal (Simpson et al. 2005). A figure of 0.8 means that ordinary people in early to mid adulthood would be prepared to sacrifice 20% of their life expectancy to be cured of CF (time trade-off), or to accept a 20% chance of death to be cured of CF (standard gamble).

In short, the bad effects of CF are quite significant. It is doubtful that the risks of gene editing to remove the gene for CF are as bad as a 20% chance of death, even with the risks of off-target mutations.

Applied to the case of CF, the implications of the Two-Tier View seem substantially more plausible than those of either the Comparative View or the Impersonal View. According to the Comparative View, there is a strong reason to prevent CF by gene editing but no reason to prevent it by embryo selection. This implication is not only intuitively implausible but also conflicts strongly with the popular position. The Impersonal View, by contrast, supports the popular view. It implies that there is no moral difference between preventing CF by selecting an embryo without this genetic disorder and preventing it by editing out the gene that causes it - even though the failure to do the latter would be worse for the child born with CF whereas the failure to do the former would not be. If this were correct, then it would be wrong to use gene editing whenever it would involve any risk, however small. For in both cases the impersonal reason to avoid the disorder is equally strong, so any risk associated with gene editing tips the balance in favor of selection.

As we have suggested, however, there are instances in which it seems to make a moral difference whether a bad effect is worse for a person. Suppose, for example, that one can either save a 60-year-old person, thereby enabling that person to live to 80, or one can cause a person who will live to 80 to exist rather than allowing a person who would live only to 60 to come into existence instead. The Impersonal View, which entails the No-Difference View, implies that the reasons to do these acts are equally strong. But most of us believe that the reason to save the one person’s life is stronger, for the failure to save that person would be worse for her (McMahan 2020). The Impersonal View also implies that there is no moral difference between selecting an embryo with the CF gene, which would not be worse for the child born with CF, and causing a child to have rather than not have CF by “editing in” the CF gene, which *would* be worse for the child born with CF. This too seems counterintuitive.

Not only do the Impersonal View and the associated No-Difference View have implausible implications about choices between gene editing and embryo selection, but the Impersonal View is also arguably incompatible with a founding principle of clinical genetics and genetic counseling.

This principle is that that genetic counseling should be “non-directive.” That is, it should simply explain the options to people and then allow them to choose for themselves. The aim of screening for Down Syndrome, for example, is said to be only to offer “choice.” The idea is that couples should be free to choose whether to have children, when to have them, how many to have, and whether they should avoid having those who would have certain characteristics or conditions. This is required by respect for reproductive autonomy.

The screening or testing of embryos and fetuses is seen as an option that might be offered to couples, but not one that should be recommended to them or urged on them. And couples should not be blamed or held accountable for not choosing it, or for choosing to continue with a pregnancy when a problem with the embryo has been discovered or is suspected. Of course, offering a person advice is generally fully compatible with respect for that person’s autonomy. But the advice of genetic counselors, like the advice of physicians, tends to be regarded as authoritative, so that those who defy it may doubt their own judgments without sufficient reason to do so, or may experience inappropriate guilt if the advice happens on this occasion to turn out to be correct, or may simply feel coerced to accept the counselor’s judgment. Hence the insistence on non-directive counseling.

This insistence is consistent with the Comparative View *if* screening and selection would be done before one of us has begun to exist—that is, before the embryo or fetus would be identical with the person who might develop from it. According to the Comparative View, the only moral reasons to cause or not to cause people to exist are those concerned with effects on people other than those who might be caused to exist—which, of course, is highly implausible. If, however, screening and any subsequent action (such as gene editing or abortion) based on the findings would be done *after* the embryo or fetus had become identical with the later person, then the implications of the Comparative View are presumably inconsistent with non-directive counseling. For it is implausible to suppose that claims to reproductive freedom, or freedom from unintended coercion, make it permissible to allow one’s children to have serious genetic disorders when those same children could be prevented from having them. This suggests yet another reason to reject the Comparative View—namely, that it implies that there is no moral objection, other things being equal, to selecting an embryo with a genetic disorder just before the individual who will be identical with the later person begins to exist, but also implies that there is a strong reason to use gene editing to eliminate that same disorder immediately after that individual has begun to exist—even if it involves a significant risk of causing a different, lesser disorder.

Like the Comparative View, the Two-Tier View is incompatible with the popular position, as it too recognizes and often gives priority to person-affecting reasons. It thus implies that gene editing may be morally required because it can prevent people from having serious disorders, or cure them of these disorders. And, because it recognizes impersonal reasons as well, it also challenges the insistence on non-directive genetic counseling in cases of embryo selection.

It is only the Impersonal View that is consistent with the popular position on the priority of selection over editing, as it implies that the impersonal reasons to practice embryo selection and gene editing are of equal strength when the outcomes of both practices would be the same. This means that, in cases in which selection and editing are both possible, editing is wrong whenever it has risks that selection does not have.

But the Impersonal View is deeply inconsistent with both moral intuition and current practice. It implies, as we saw in the case of CF, that selecting an impaired or diseased embryo is as wrong as deliberately causing an embryo, fetus, or infant to be impaired or diseased. It thus implies that selecting a deaf embryo is as seriously objectionable as deafening an infant by severing the auditory nerves. The Impersonal View is also incompatible with the orthodox view of genetic counseling. For it implies that the reason to use embryo selection to avoid causing a serious genetic disorder is as strong as the reason to cure that same disorder in an existing child. And a reason of this latter strength is unlikely to be overridable by rights of reproductive autonomy or claims against inadvertent coercion. Although we think the Impersonal View is correct to reject non-directive genetic counseling, the Two-Tier View has the same desirable implication.

## AN OBJECTION

We have thus far argued that, in causing a person to have a genetic disorder, gene editing is morally worse than embryo selection. This is because “editing in” a genetic disorder, such as CF, is worse for the subsequent person, whereas selecting an embryo with the disorder is not only not worse for the subsequent person but is, if the person will have a life worth living, good for that person. And we have suggested that, by parity of reasoning, “editing out” a genetic disorder is, in one respect, morally better than selecting an embryo that does not have that disorder. This is because editing out the disorder would be better for the subsequent person, whereas selecting the unaffected embryo would not be.

This reasoning has been criticized by several philosophers on roughly the following ground. The claim that gene editing was better for the person who developed from the beneficently edited embryo implies that, if the editing had not been done, that would have been worse for the person. But if, when the person’s parents were deliberating about whether, when, and how to have a child, they had chosen embryo selection rather than gene editing, gene editing would not have been done, but that would not have been worse for the person who in fact became their child; for their choice of embryo selection would have ensured that their actual child would never have existed (Rulli 2019; Sparrow 2022; Douglas and Devolder 2022).

This objection derives from a simple fact: that when an act is done that causes a certain effect, there are often indefinitely many ways in which it could have been true that the act was not done and thus equally many alternative courses of events that might have occurred instead of the actual course of events. Suppose, for example, that beneficent gene editing has been successfully done. The many ways in which it might not have been done include the following. The parents might have decided to remain childless. They might have produced several embryos via IVF and selected one without a genetic disorder (or one with a genetic disorder). They might have produced one embryo intending to use gene editing but then decided to discard it. They might have produced one embryo and discovered that it had the disorder but implanted it without doing the gene editing. Or they might have produced one embryo with the disorder, used gene editing to cause a further disorder, and then implanted it. The last two of these alternatives would have been worse for the person who in fact developed from the edited embryo. But the first three would not have been.

No one of these alternatives provides the uniquely correct comparison for determining whether the actual gene editing was better for the person. Some think that the correct alternative for determining whether an act was better or worse for someone affected by it is what *would* have been done otherwise. But there are still various alternative acts that would have been done—just at different times. It might be, for example, that when the parents were deciding how to produce a child, they would have chosen embryo selection if they had not chosen gene editing. Then, later, once the embryo had been produced, it might be that they would have discarded the embryo had they not edited and implanted it.

Furthermore, what would otherwise have been done at a particular time does not always determine whether what was actually done at that time was better or worse for a person affected by it. Suppose, for example, that an embryo was created, found to have a genetic disorder, but was then implanted without gene editing, even though gene editing was possible. We can say unequivocally that this was worse for the person who now has to live with the disorder. And this is true even if, for example, the parents had a religious objection to gene editing (though not to discarding an embryo), so that they *would* have discarded the embryo if they could not have implanted it without its being edited. Even so, their child has a justified complaint against them. “Who cares,” he might say, “what they would have done if they had not implanted the unedited embryo from which I developed. What matters is that I have got a terrible disorder and they *could* have enabled me to be without it.”

It is clearly worse, then, for a person to have a genetic disorder because the embryo from which the person developed was implanted without gene editing when gene editing was feasible. The implicit, normatively salient alternative was for the disorder to be eliminated by gene editing—irrespective of whether that would in fact have been done. That implantation without gene editing was worse for the person implies that gene editing would have been better for that person. It therefore seems that, whenever gene editing has successfully been done, that was better for the person who has developed from the edited embryo even if the embryo would have otherwise been discarded rather than implanted without editing—and even if, much earlier, the parents would have chosen embryo selection had they not chosen gene editing.

By contrast, when embryo selection has been done and has resulted in the existence of a person without a genetic disorder, that is *never* ascertainably better for the person than if the selection had not been done. For there was at no point any alternative to the selection of that embryo—for example, deciding not to have a child, choosing gene editing rather than embryo selection, selecting a different embryo, and so on—that would have been foreseeably worse for the person who developed from that embryo. It is possible, though highly improbable, that the parents could have made a different choice at some point that would nevertheless have resulted in the existence of the same person, though because the circumstances of the person’s coming into existence would have been different, the person would have had a different and worse life. But this would have been entirely fortuitous and unpredictable and is thus irrelevant to the question whether embryo selection is ever foreseeably better for the person who will develop from the selected embryo.

We will conclude this section by briefly addressing a further argument for the claim that gene editing is actually identity determining. Robert Sparrow has argued that, for gene editing to be successful, it would be necessary to create a number of embryos, edit each one, use preimplantation genetic diagnosis to determine in which the editing has been most successful without introducing unwanted mutations, and then implant that best embryo (Sparrow 2022). In this case, the child who develops from the most successfully edited embryo cannot claim that the editing was better for her, as the relevant alternative is that a different embryo would have been implanted and the actual child would never have existed.

Our response to this objection is again to appeal to the parallel case of inserting a disadvantageous gene. Suppose that a couple, for whatever reason, want to have a child with a genetic disorder. They go through the process described by Sparrow, except that they select and implant the edited embryo that has the most severe form of the disorder. They would, however, have implanted a different one if there had been another edited embryo with an even more severe form of the disorder. But this does not mean that what they have done is not worse for their actual child with the disorder. That child can reasonably complain that her parents have caused her to have the disorder when, had they acted differently, she could have existed without it. They cannot, it seems, rebut that complaint by contending that what they did was actually good for their child (though not better) because, had they not successfully inserted the gene for the disorder into the embryo from which she developed, she would never have existed, since they would have selected a different, more successfully edited embryo instead. Even if that is what they would in fact have done, it remains true that their child is worse off than she could have been if they had acted differently. And it adds to the seriousness of her complaint that they have caused her to be worse off for reasons of self-interest. But if the insertion of a disadvantageous gene in these conditions is worse for the person who has it, then the removal of the same gene in parallel circumstances is better for the person who lacks it.

## PLEIOTROPY

There are certain types of case in which embryo selection may be morally better than gene editing. One involves either editing out or inserting genes that are pleiotropic. Genes of this sort can increase the likelihood that an individual will have two or more quite different traits. Some such genes have been carefully studied: for example, genes associated with “same-sex sexual behaviour” have been found to be also associated with a disposition to have more sexual partners (possibly by increasing risk taking and openness to experience), thus conferring “a mating advantage” when present in individuals who engage in “opposite-sex sexual behaviour” (Zietsch et al. 2021).

The association between artistic creativity and bipolar disorder may also be an instance of pleiotropy—though this has been less well studied (Jamison 1993). Let us suppose, for the sake of argument, that the same gene or genes dispose their possessors to both creativity and bipolar disorder. If the reason not to cause harm is in general stronger than the reason to confer a benefit of equivalent magnitude, then in cases in which the same gene may cause both beneficial and harmful effects, there may be a presumption against an attempt at enhancement through “editing in” that gene (Savulescu et al. 2006).

Consider two different couples. Couple 1 have two embryos. The first has a disposition to bipolar disorder and creativity. The second has neither but is otherwise similar. If, because they want their child to be creative, they select the first and the child has a life pervaded with severe mental illness but little ability to exercise creativity, they will have made the wrong decision. But what they have done is not worse for the child.

Couple 2 have one embryo. They would like to edit it to increase their child’s creative talents, though they know that this would increase the child’s risk of developing bipolar disorder. If they do engage in gene editing and the child then suffers significantly from bipolar disorder with little creativity, they will have done what is worse for the child (assuming that the editing was identity-preserving). For the child can reasonably complain that if her parents had not tampered with the embryo from which she developed, she would not be burdened with mental illness. This seems true even if what the parents would in fact have done if they had not engaged in editing would have been to discard the embryo and then engage in embryo selection. Even so, their actual child can still reasonably complain that “I could have lived without this burden but they had to have a little artistic genius.”

Since the Two-Tier View asserts that causing a bad effect that is worse for someone is more seriously wrong than causing an equivalent bad effect that is not worse for anyone, it implies that Couple 2’s action is more seriously wrong than Couple 1’s. In these cases, editing is worse than selection.

Selection cannot be worse in person-affecting terms, even when the embryo selected will have a life that is not worth living (for, as we have noted, although such a life is *bad* for the individual whose life it is, it is not *worse* for that individual). If we are uncertain either about the value of certain traits or about the probability of the contribution to traits, selection may be morally less risky than gene editing (Gyngell, Douglas, and Savulescu 2017).

## CONCLUSION

Among the many implausible implications of the Comparative View is the claim that, while there is a strong moral reason to use gene editing to eliminate a genetic disorder, there is no reason, apart from reasons concerned with harmful side effects on others, to use embryo selection to avoid the same disorder (unless we begin to exist at conception or very soon thereafter). By contrast, both the Impersonal View and the Two-Tier View recognize that there are moral reasons to use both embryo selection and gene editing. According to the Impersonal View, these reasons are of equal strength, other things being equal. But so are the reasons not to *cause* the same genetic disorder by these means. The impersonal reason not to insert the gene for CF by gene editing is, on this view, no stronger than the reason not to select an embryo with CF rather than one without it, even though the former would be worse for the subsequent person whereas the latter would be good for the resulting person. That seems implausible to many of us.

The Impersonal View is the only view of reproductive reasons that is consistent with the popular position on gene editing and embryo selection. Yet it is inconsistent with common practice, in that it implies that testing and selection, and thus directive counseling, are morally required (as does the principle of Procreative Beneficence; see Savulescu 2001). While this may not be implausible, it does seem clearly implausible to claim, as the Impersonal View does, that the failure to avoid a genetic disorder by failing to use embryo selection is as seriously wrong as failing to treat, or even causing, the same disorder in an existing person.

Unlike the Impersonal View, the Two-Tier View recognizes that the reason not to use gene editing (or some alternative means) to cause a person to have a disorder, such as CF, when that person would otherwise not be afflicted with it, is stronger than the reason not to select an embryo with the disorder rather than one without it. But it also implies that the reason to use gene editing to prevent a person from having such a disorder when the person would otherwise have it is, in some instances at least, stronger than the reason to select an embryo without the disorder rather than one with it.

Finally, because the Two-Tier View recognizes impersonal reasons as well as person-affecting reasons, it implies that there is a further reason to pursue techniques of gene editing, which is that this will facilitate the development not only of techniques for safely removing genetic disorders from the human genome but also of techniques for safely enhancing the human genome, thereby enabling our successors to have lives that are better than the best of which we are capable.

As we have acknowledged, gene editing involves a risk of causing unforeseen, disadvantageous mutations. These would be bad for the person who would develop from the edited embryo. But it is difficult to see how such a mutation could be *worse for* the subsequent person unless the intended removal of the bad gene or genes would be *better for* that same person. (If one thinks, for example, that the relevant alternative to the gene editing is the discarding of the embryo, then neither the beneficent editing nor the unintended mutation would be worse for the subsequent person.) So, if the disorder edited out would be worse than the mutation accidentally caused, then the gene editing would still be on balance better for the person who would develop from the embryo. If that person’s life would nevertheless be less good than that of a person who would have come into existence if embryo selection had been used rather than gene editing, then the use of gene editing would be worse impersonally. But if, over time, the use and further development of techniques of gene editing would enable scientists to develop techniques of germ-line genetic enhancement that they would otherwise not be able to develop, or that they would otherwise be able to develop only later, then it is likely that the more general use of gene editing rather than embryo selection would be overall better both impersonally and in person-affecting terms.^1^

## Footnotes

1 Those who accept the Two-Tier View can differ about *how much* it matters that a bad effect is worse for someone rather than not worse for anyone. Both of us accept that this difference matters to a significant degree in cases in which the individual who is affected for the worse is an adult person with strong interests. This is true, for example, in the comparison cited between the killing of a 60-year-old and the choice to cause a shorter-lived person to exist rather than allow a different, longer-lived person to come into existence. Yet in the cases with which we are primarily concerned in this essay – cases involving editing the genes of an embryo – we have assumed that the individual who might be affected for the worse does not yet exist at the time of choice and thus has no present interest in having the healthier life. Because of this, one of us – McMahan – thinks that the fact that this individual would be affected for the worse by having the less good life matters less. The argument for this claim is complex and cannot be given here. (See McMahan 2002, chapter 1; and McMahan 2023.) But what this means is that McMahan thinks that the moral differences between embryo selection and gene editing are less important than Savulescu thinks they are.
